# Factors associated with survival of patients with solid Cancer alive after intensive care unit discharge between 2005 and 2013

**DOI:** 10.1186/s12885-020-07706-3

**Published:** 2021-01-05

**Authors:** Hubert Gheerbrant, Jean-François Timsit, Nicolas Terzi, Stéphane Ruckly, Mathieu Laramas, Matteo Giaj Levra, Emmanuelle Jacquet, Loic Falque, Denis Moro-Sibilot, Anne-Claire Toffart

**Affiliations:** 1 Department of Pneumology and Physiology, Grenoble-Alpes University Hospital, BP217, FR-38043 Grenoble Cedex 9, France; 2 Medical and Infectious Diseases ICU, APHP, Paris, France; 3 Department of Biostatistics, OUTCOMEREA™, Bobigny, France; 4 Department of Intensive Care and Reanimation, Grenoble-Alpes University Hospital, Grenoble, France; 5 INSERM, U1042, Grenoble-Alpes University Hospital, HP2, Grenoble, France; 6 Cancer and Blood Diseases, Grenoble-Alpes University Hospital, Grenoble, France; 7 Institute for Advanced Biosciences – INSERM U823 – Grenoble-Alpes University, Grenoble, France

**Keywords:** Solid cancer, Intensive care unit, Prognosis, Anticancer treatments, Performance status, Survival

## Abstract

**Background:**

At intensive care unit (ICU) admission, the issue about prognosis of critically ill cancer patients is of clinical interest, especially after ICU discharge. Our objective was to assess the factors associated with 3- and 6-month survival of ICU cancer survivors.

**Methods:**

Based on the French OutcomeRea™ database, we included solid cancer patients discharged alive, between December 2005 and November 2013, from the medical ICU of the university hospital in Grenoble, France. Patient characteristics and outcome at 3 and 6 months following ICU discharge were extracted from available database.

**Results:**

Of the 361 cancer patients with unscheduled admissions, 253 (70%) were discharged alive from ICU. The main primary cancer sites were digestive (31%) and thoracic (26%). The 3- and 6-month mortality rates were 33 and 41%, respectively. Factors independently associated with 6-month mortality included ECOG performance status (ECOG-PS) of 3–4 (OR,3.74; 95%CI: 1.67–8.37), metastatic disease (OR,2.56; 95%CI: 1.34–4.90), admission for cancer progression (OR,2.31; 95%CI: 1.14–4.68), SAPS II of 45 to 58 (OR,4.19; 95%CI: 1.76–9.97), and treatment limitation decision at ICU admission (OR,4.00; 95%CI: 1.64–9.77). Interestingly, previous cancer chemotherapy prior to ICU admission was independently associated with lower 3-month mortality (OR, 0.38; 95%CI: 0.19–0.75). Among patients with an ECOG-PS 0–1 at admission, 70% (*n* = 66) and 61% (*n* = 57) displayed an ECOG-PS 0–2 at 3- and 6-months, respectively. At 3 months, 74 (55%) patients received anticancer treatment, 13 (8%) were given exclusive palliative care.

**Conclusions:**

Factors associated with 6-month mortality are almost the same as those known to be associated with ICU mortality. We highlight that most patients recovered an ECOG-PS of 0–2 at 3 and 6 months, in particular those with a good ECOG-PS at ICU admission and could benefit from an anticancer treatment following ICU discharge.

**Supplementary Information:**

The online version contains supplementary material available at 10.1186/s12885-020-07706-3.

## Background

In 2018, the World Health Organization (WHO) estimated the number of new cancer occurrences at 18.1 million worldwide, and the specific cancer-related mortality at about 10 million. In addition, the WHO estimates that the global cancer incidence would increase by more than 63% in 2040 as compared to 2018 [1]. Furthermore, improvements in anticancer treatments have improved the overall survival, which is associated with a significant increase of cancer prevalence worldwide [2, 3]. Nevertheless, improving patients’ life expectancy does not exclude their fragility, given that approximately 5–10% of them will develop a life-threatening disease requiring intensive care unit (ICU) admission [4].

This is an issue facing intensive care physicians, both in terms of the ICU admission of these patients, as well as their management. These patients represent 15–20% of all ICU admissions [5–8]. Despite therapeutic improvements for cancer patients, an ICU admission is still associated with a very poor medium-term prognosis [9, 10]. While intra-hospital mortality is estimated around 25–35% [5, 6, 11], with no significant difference as compared to patients without cancer [11–13], the 1-year mortality often exceeds 70% [14–16]. Prognostic factors associated with ICU survival are related to cancer, general condition of the patient and acute disease. Regarding cancer characteristics, extension of cancer and eligibility of the patient to an anticancer treatment are more important than type or histology of the cancer [17]. A better understanding of these prognostic factors associated has been associated with an improved patient selection upon ICU admission [4, 18, 19]. However, while the factors associated with patients’ being still alive at ICU discharge are much less known, they are likewise less taken into account at admission [17]. Thus, it appears necessary to better understand these factors in order to better identify the cancer patients that most likely could benefit from the ICU stay [20]. The mortality has proven to be largely associated with the general patient condition (Eastern Cooperative Oncology Group performance status [ECOG-PS]) at ICU discharge [10, 21]. This association can be partly explained by the fact that the cancer management strategy is dependent on a patient’s ECOG-PS conditions [22, 23]. To our knowledge, the evaluation of the oncologic management pertaining to these patients discharged alive from intensive care has not been fully assessed.

This research work sought to further determine the factors associated with the survival of cancer patients still alive at ICU discharge. We also sought to describe their general condition and anticancer treatments following their ICU stay.

## Methods

### Design and setting

We conducted a retrospective analysis involving a French multicenter prospective observational cohort entered into the OutcomeRea™ database previously described [24]. The database is fed by 12 French ICUs, and contains data on admission features and diagnosis, daily disease severity, iatrogenic events, nosocomial infections, and vital status. In some cases, participants in the OUTCOMEREA group have enrolled consecutive patients admitted to ICU, and in others sampling has been performed among all consecutive admissions during a period of time or all admissions to certain ICU beds. Data included in the OUTCOMEREA database have been collected by senior physicians or research monitors of the participating ICUs. For each patient, the data were first entered into an electronic case-report form using e-RHEA data-capture software (OUTCOMEREA, Drancy, France), and all case-report forms were then entered into the OUTCOMEREA data warehouse. At entry in the database, the data-capture software automatically conducts multiple checks for internal consistency of most of the variables. Queries generated by these checks were resolved with the source ICU before incorporating the new data into the database. A 1-day coding course is organized annually with the study investigators and clinical research monitors.

Study ethics approval was obtained on 09 October 2019 (Ethics Committee of Clinical Investigation Centers of Rhône-Alpes-Auvergne, Clermont-Ferrand, IRB 5891). An information letter was sent to each living patient providing him the opportunity to refuse study participation.

The primary objective was to identify the factors associated with 3- and 6-month mortality after ICU discharge. The secondary objectives were to assess the ECOG-PS and anticancer treatments at 3- and 6-months.

### Study population

We included solid tumor patients admitted, between December 2005 and November 2013, to the medical ICU of the Grenoble Alpes University Hospital in France. Patients were retrieved from the OutcomeRea™ database, and we selected only those with an International Statistical Classification of Diseases by the World Health Organization (ICD-10) related to solid tumor (C00 to C97). Exclusion criteria were patients under 18 years of age at admission, cancer in remission for over 5 years, hematological malignancy, lack of histological or cytological cancer diagnosis upon ICU admission, programmed hospitalization for post-surgery or central venous line placement, as well as referral from another ICU. Two different admissions for the same patient were independently considered, provided that they were separated by more than 3 months. If they were closer, only the first was taken into account.

### Data collection

Data related to both the ICU admission and stay were extracted from the OutcomeRea™ database. Complementary data relating to cancer history before and at 3- and 6-months following ICU admission were retrieved from the patients’ computerized medical charts. Primary tumor sites were defined as digestive (gastrointestinal, esophageal, liver, and pancreas), thoracic (lung and mesothelioma), head and neck, genitourinary (including testicles), gynecological (including breast), and other (endocrine, skin, brain, sarcoma, and rare cancers). Other cancer-related data retrieved were: metastatic status at ICU admission, time from diagnosis, anticancer treatments, and cancer status at the last oncological evaluation (newly diagnosed or in recurrence, controlled or in remission for less than 5 years, progression). At admission, we recorded the ECOG-PS [25], comorbidities using the Charlson comorbidity index [26], reason for admission (thrombotic event, bleeding, complications of oncology therapy, or not cancer-related), sepsis-related organ failure assessment (SOFA) score [27], as well as simplified acute physiologic score II (SAPS II) [28]. Treatments applied within the ICU were also collected (vasoactive drugs, invasive mechanical ventilation, or renal replacement therapy), along with potential limitations regarding care decisions. Data regarding ECOG-PS and new anticancer treatments administered were collected at 3- and 6-months following ICU discharge. Survival at 3- and 6-month were completed for all patients by either consulting the hospital medical chart or providing a call to the place of birth.

### Statistical analysis

Characteristics of patients were described as median (interquartile range) or number (percent) as appropriate. The SAPS II and SOFA scores have been expressed in points. Three- and 6-month survivals were defined as patients alive at 3 and 6 months from ICU discharge. Patients lost to follow-up at 3 or 6 months were considered as missing data.

Univariate logistic regression models were used to investigate potential risk factors of 3- and 6-month mortality. Multivariate logistic regressions were used to assess risk factors. The following clinically relevant variables were forced in the multivariate models (i.e.*,* well-established risk factors for death at 3 and 6 months): ECOG-PS, Metastatic disease, Previous anticancer treatment: Chemotherapy, Reason for ICU admission: cancer progression, SAPS II and TLD before ICU discharge. Ten missing values for ECOG-PS and 11 missing values for Metastatic disease were imputed to the mod in the multivariate models. Linearity to the logit for continuous variables was checked with Generalized Additive Models, non-linear variables were categorized according to quartiles. Results were expressed as odds ratios (ORs), with 95% confidence intervals (CIs) and *P* values.

All tests were two-sided, and *P* values < 0.05 were considered statistically significant. All statistical analyses were performed using SAS 9.4 (SAS Institute, Cary, NC, USA).

## Results

### Patient characteristics

**Fig. 1:**
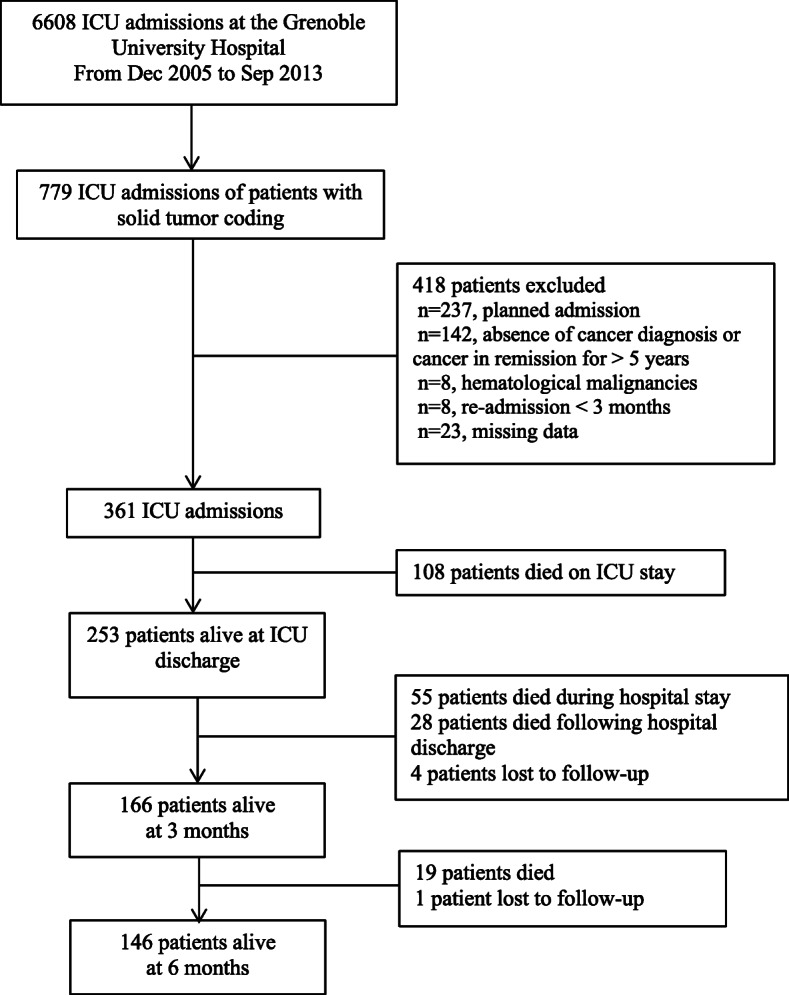
Patient Flow chart. ICU = intensive care unit

Of the 6608 patients admitted between December 2005 and November 2013 to the Grenoble ICU and recorded into the OutcomeRea™ database, 779 concerned cancer patients (Fig. 1). After considering inclusion and exclusion criteria, 361 ICU admissions were selected. ICU mortality was 30% (*n* = 108), resulting in 253 studied patients. The median follow-up following ICU discharge was 250 days (IQR 25–75%: 41–748). Four patients were lost to follow-up at 3 month and 5 at 6 month.

Patient characteristics have been reported in Table 1. The main primary tumor sites were digestive (*n* = 79, 31%) and thoracic (*n* = 65, 26%). Almost half of the patients (*n* = 108, 45%) had a metastatic disease at ICU admission and 167 (68%) patients displayed a newly diagnosed cancer or cancer in progression. Most patients (*n* = 149, 59%) were treated by chemotherapy prior to ICU admission. The ECOG-PS at admission was lower or equal to 2 for 192 (76%) patients. The main reasons for ICU admission were tumor progression in 60 (24%) patients and not cancer-related in 117 (46%).

**Table 1:** Main Patient Characteristics at admission and during ICU stay

Variable	***n*** = 253
**Patient Characteristics**	
Female gender	77 (30)
Age (years)	64 [55–71]
ECOG-PS (miss. = 10)	
0–1	114 (47)
2	78 (32)
3–4	51 (21)
Charlson comorbidity index	1 [0–3]
**Cancer Characteristics**	
Type of cancer	
Digestive	79 (31)
Thoracic	65 (26)
Head and Neck	32 (13)
Gynecological	29 (12)
Genito-urinary	28 (11)
Other	23 (9)
Metastatic disease (miss. = 12)	108 (45)
Previous anticancer treatment	
Surgery	119 (47)
Radiotherapy	81 (32)
Chemotherapy	149 (59)
Cancer status (miss. = 6)	
Controlled or in remission for < 5 years	80 (32)
Newly diagnosed / recurrence	102 (41)
In progression	65 (26)
**ICU Characteristics**	
Reason of ICU admission^a^	
Tumor progression	60 (24)
Thrombotic event	18 (7)
Bleeding	28 (11)
Complications of anticancer treatment	61 (24)
Not related to cancer	117 (46)
SAPS II	46 [36–58]
Vasopressors	88 (34.8)
Invasive ventilation	103 (40.7)
Renal replacement therapy	22 (9)
TLD before ICU discharge	40 (16)
Length of stay in ICU (days)	4 [2–9]

Qualitative variables are expressed as n (%) and quantitative variables as median (interquartile range 25–75%)

*ICU* intensive care unit, *OR* odds ratio, *miss.* missing data, *PS* performance status, *TLD* treatment limitation decision

^a^Variables not mutually exclusive

The median ICU length of stay was 4 days [IQR 25–75%, 2–9]. Upon their ICU stay, the decision to withhold or withdraw life-sustaining treatments was made for 40 (16%) patients.

### Outcome analyses

After ICU discharge, the median hospital stay length was 12 days [IQR 25–75%, 5–23], and the median survival was 173 days [IQR 25–75%, 18–622]. The 3- and 6-month mortality rates were 33% (*n* = 83/249, 4 lost to follow-up) and 41% (*n* = 102/248, 5 lost to follow-up), respectively (Fig. 1).

The univariate analysis results aimed to identify factors associated with 3- and 6-month mortality are listed in Table 2. SOFA variables were not included because they were collinear with SAPS II. The multivariate analysis results are displayed in Table 3. The main determinants of 3- and 6-month mortality were an ECOG-PS of 3 or 4, metastatic disease at ICU admission, ICU admission for cancer progression, and treatment limitation decision taken within the 2 days preceding ICU discharge. Having been treated with chemotherapy prior to ICU admission was associated with an improved 3-month survival. A high SAPS II was only associated with 6-month mortality.

**Table 2:** Main Patient Characteristics Associated With 3- and 6-Month Mortality From Intensive Care Unit Admission in Patients Who Were Discharged Alive From ICU (Univariate Analysis)

Variable	At 3 Months ***n*** = 249* (83 deaths)		At 6 Months ***n*** = 248* (102 deaths)	
	OR (95%CI)	***p***-value	OR (95%CI)	***p***-value
**Patient Characteristics**				
Gender Female (ref = male)	1.12 (0.63–1.99)	0.69	0.91 (0.52–1.59)	0.75
Age (per year)	1.02 (1–1.04)	0.13	1.01 (0.99–1.04)	0.17
ECOG-PS (miss. = 10)		< 0.01		< 0.01
- 0–1	1.00		1.00	
- 2	1.83 (0.96–3.5)		2.12 (1.15–3.9)	
- 3–4	4.86 (2.38–9.95)		5.07 (2.48–10.39)	
Charlson comorbidity index (per point)	1.02 (1–1.03)	0.80	0.94 (0.82–1.09)	0.44
**Cancer Characteristics**				
Type of cancer				
- Digestive (ref = absence)	1.22 (0.69–2.14)	0.50	1.05 (0.61–1.82)	0.86
- Thoracic (ref = absence)	1.49 (0.83–2.67)	0.19	1.9 (1.07–3.36)	0.03
- Head and Neck (ref = absence)	0.42 (0.17–1.06)	0.07	0.38 (0.16–0.92)	0.03
- Gynecological (ref = absence)	1.13 (0.49–2.56)	0.78	1.1 (0.5–2.45)	0.81
-Genito-urinary (ref = absence)	1.0 (0.43–2.33)	1	0.7 (0.3–1.63)	0.41
- Other (ref = absence)	0.68 (0.26–1.80)	0.44	0.93 (0.39–2.24)	0.87
Metastatic disease (miss. = 12) (ref = absence)	2.29 (1.33–4)	< 0.01	2.46 (1.45–4.16)	< 0.01
Previous anticancer treatment				
- Surgery (ref = absence)	0.60 (0.35–1.02)	0.06	0.60 (0.36–1)	0.05
- Radiotherapy (ref = absence)	0.71 (0.4–1.27)	0.25	0.86 (0.5–1.48)	0.58
- Chemotherapy (ref = absence)	0.71 (0.42–1.21)	0.20	1 (0.6–1.67)	0.99
Cancer status (miss. = 6)		0.19		0.03
- Controlled or in remission for < 5 years	1.00		1.00	
- Newly diagnosed / recurrence	1.41 (0.73–2.69)		1.65 (0.88–3.08)	
- In progression	1.92 (0.95–3.89)		2.57 (1.29–5.11)	
**ICU Characteristics**				
Reason of ICU admission				
Tumor progression (ref = absence)	2.67 (1.47–4.88)	< 0.01	2.72 (1.49–4-95)	< 0.01
- Thrombotic event (ref = absence)	1.66 (0.63–4.39)	0.30	2.44 (0.91–6.54)	0.08
- Bleeding (ref = absence)	1.13 (0.49–2.56)	0.78	1.1 (0.5–2.45)	0.81
- Complications of anticancer treatment (ref = absence)	0.84 (0.45–1.59)	0.60	1.14 (0.63–2.06)	0.67
- Not related to cancer (ref = absence)	0.53 (0.31–0.91)	0.02	0.4 (0.24–0.68)	<.01
SAPS II (per point)	1.02 (1–1.03)	0.03	1.02 (1.0–1.03)	0.02
Supportive Care				
- Vasopressors (ref = absence)	1.17 (0.68–2.03)	0.57	1.04 (0.61–1.77)	0.88
- Invasive ventilation (ref = absence)	0.8 (0.46–1.37)	0.41	0.78 (0.47–1.31)	0.35
- Renal replacement therapy (ref = absence)	0.93 (0.36–2.37)	0.87	0.82 (0.33–2.03)	0.66
TLD before ICU discharge (ref = absence)	6.53 (3.11–13.74)	< 0.01	3.22 (1.25–8.29)	< 0.02
Length of stay in ICU (per day)	0.99 (0.96–1.02)	0.42	0.99 (0.96–1.01)	0.30

*Five patients were lost to follow-up at 1, 30, 62, 64 and 129 days, respectively. They were excluded for the analyses

*CI* confidence interval, *ICU* intensive care unit, *OR* odds ratio, *miss.* missing data, *PS* performance status, *TLD* treatment limitation decision

**Table 3:** Multivariate Analysis of Characteristics Associated With 3- and 6-Month Mortality

Variable	At 3 Months ***n*** = 249* (83 deaths)		At 6 Months ***n*** = 248* (102 deaths)	
	OR (95%CI)	***p***-value	OR (95%CI)	***p***-value
**ECOG-PS**		0.006		0.006
0–1	1		1	
2	1.24 (0.60–2.54)		1.42 (0.73–2.79)	
3–4	3.67 (1.62–8.34)		3.74 (1.67–8.37)	
**Metastatic disease**	2.74 (1.38–5.44)	0.004	2.56 (1.34–4.90)	0.004
**Previous anticancer treatment**				
Chemotherapy	0.38 (0.19–0.75)	0.006	0.61 (0.32–1.16)	0.13
**Reason for ICU admission**				
Cancer progression	2.08 (1.02–4.23)	0.04	2.31 (1.14–4.68)	0.02
**SAPS II**		0.08		0.01
[0–35]	1		1	
[35–45]	2.74 (1.06–7.08)		2.65 (1.09–6.47)	
[45–58]	3.15 (1.26–7.89)		4.19 (1.76–9.97)	
[58–160]	2.68 (1.04–6.89)		2.55 (1.05–6.20)	
**TLD before ICU discharge**	4.21 (1.80–9.86)	< 0.001	4.00 (1.64–9.77)	0.002

*Five patients were lost to follow-up at 1, 30, 62, 64 and 129 days, respectively. They were excluded for the analyses

*CI* confidence interval, *ICU* intensive care unit, *OR* odds ratio, *TLD* treatment limitation decision

The median survival was 332 days [IQR 25–75%, 35–1476] in patients previously treated with chemotherapy, and 286 days [IQR 25–75%, 54–690] in those never treated with chemotherapy prior to ICU admission (Supplementary Table 1). With respect to survival curves (Supplementary Fig. 1), we observed that the curves crossed between 3- and 6-months.

### Patient presentations following ICU discharge

**Fig. 2:**
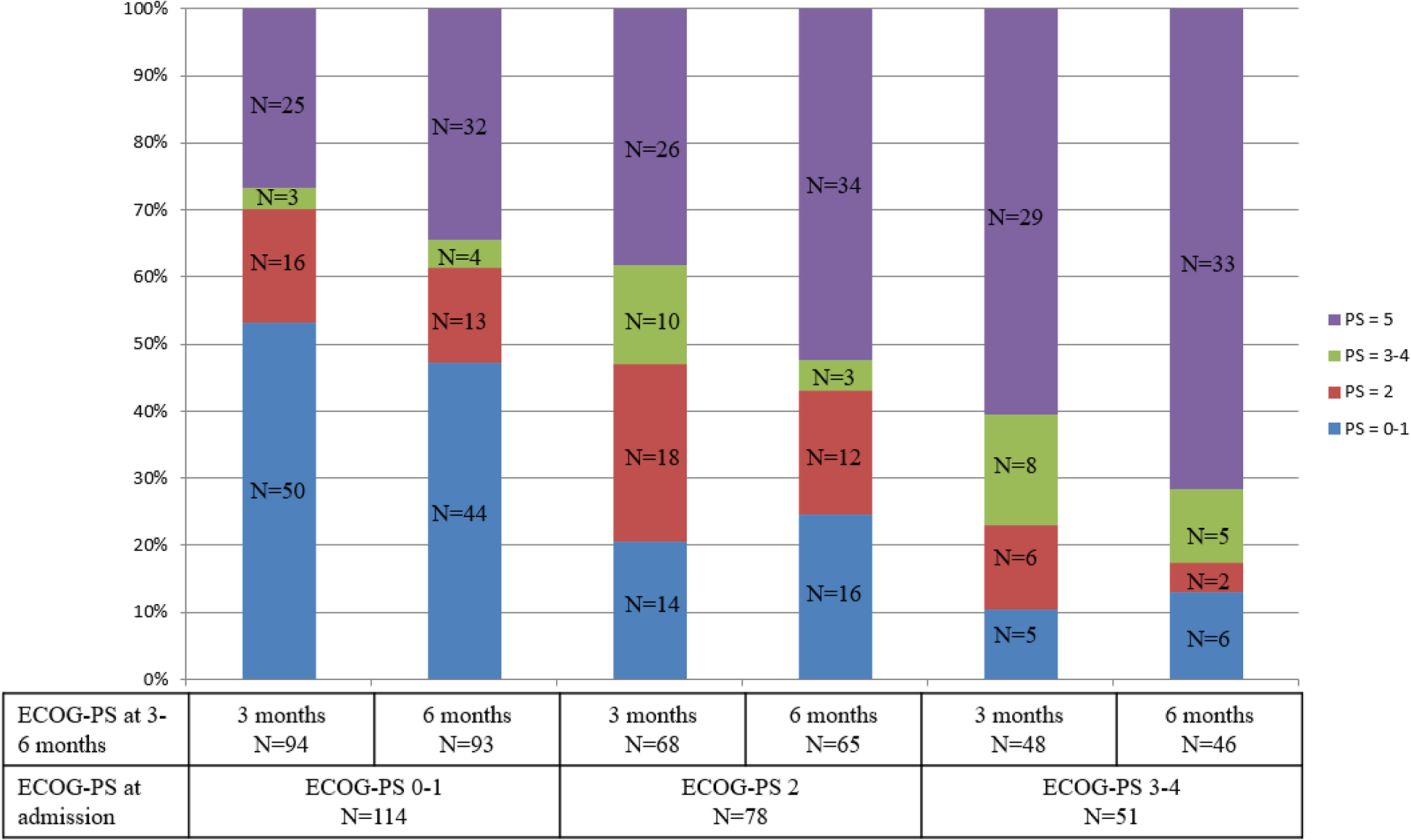
ECOG performance status at 3- and 6-months according to ECOG performance status at intensive care unit admission (40 missing data at 3 months and 46 missing data at 6 months). PS = performance status

Of the patients with an ECOG-PS of 0–1 at admission, 70% (*n* = 66) and 61% (*n* = 57) displayed an ECOG-PS of 0–2 at 3- and 6-months, respectively (Fig. 2). Only 11 (23%) and 8 (17%) patients with an ECOG-PS 3–4 at admission exhibited an ECOG-PS of 0–2 at 3 or 6 months, respectively.

At 3 months post-ICU discharge, 74 (55%) patients received anticancer treatments, while 13 (8%) were in exclusive palliative care (Table 4). The other patients had no anticancer treatment indication. Most patients with a newly diagnosed cancer, cancer recurrence (*n* = 40, 69%), or cancer in progression (*n* = 19, 68%) and still alive at 3 months were treated, mostly with chemotherapy. At 6 months, 46 (44%) patients received anticancer treatment, while 13 (9%) were in exclusive palliative care.

**Table 4:** Anticancer Treatment Following Intensive Care Unit Discharge

Variable	Total	Cancer status at admission in ICU (miss. = 3)		
		Controlled Cancer or in Remission < 5 Years	Patients With Newly Diagnosed Cancer / Recurrence	Patients With Cancer in Progression
**At 3 months**	*n* = 166	*n* = 59, miss. = 11	n = 66, miss. = 8	*n* = 38, miss. = 10
Anticancer treatment	74 (55)	14 (29)	40 (69)	19 (68)
- Chemotherapy	51 (38)	10 (21)	26 (45)	14 (50)
- Radiotherapy	17 (13)	2 (4)	15 (26)	0
- Surgery	7 (5)	0	6 (10)	1 (4)
- Other	12 (9)	4 (8)	3 (5)	4 (14)
No treatment indication	48 (29)	29 (49)	14 (21)	5 (13)
Palliative care	13 (8)	5 (8)	4 (6)	4 (11)
**At 6 months**	*n* = 146	*n* = 56, miss. = 15	*n* = 58, miss. = 15	*n* = 29, miss. = 9
Anticancer treatment	46 (44)	15 (37)	17 (40)	13 (29)
- Chemotherapy	28 (27)	7 (17)	11 (26)	9 (45)
- Radiotherapy	3 (3)	1 (2)	2 (5)	0
- Surgery	4 (4)	1 (2)	2 (5)	1 (5)
- Other	15 (14)	7 (17)	4 (9)	3 (15)
No treatment indication	46 (32)	23 (41)	20 (34)	3 (10)
Palliative care	13 (9)	3 (5)	6 (10)	4 (14)

*ICU* intensive care unit, *miss.* missing data

^a^Variables not mutually exclusive

^b^Targeted therapy, immunotherapy, hormone therapy

Concerning the 40 patients with a treatment limitation decision prior to ICU discharge, 12 (30%) were alive at 3 months, and 9 (23%) at 6 months. At 3 months, four (33%) benefited from anticancer treatment, five (42%) had no treatment indication, whereas one was in exclusive palliative care.

## Discussion

In this large mono-center study, we have reported the 3- and 6-month survival data, along with the characteristics of cancer patients discharged alive from ICU. The usual prognostic factors (ECOG-PS, metastatic disease, admission for cancer progression and treatment limitation decision) were proven to be associated with 3- and 6-month survival. Most patients with an ECOG-PS of 0–1 at ICU admission showed a good ECOG-PS (0–2) at 3 (70%) and 6 (61%) months. Of note is that 2/3 of the patients admitted in ICU with newly cancer/cancer recurrence/cancer progression and alive at ICU discharge did benefit from anticancer treatment at 3 months.

The ICU mortality of our patient cohort was 30%, thus in line with other studies [5, 11, 12, 17]. The 3-and 6-month mortality rates (33 and 41%) of patients alive at ICU discharge were likewise similar to the 90-day mortality rate reported in the *Auclin* et al. (28%) study and 120-day mortality rate from the *Vincent* et al. study (41.6%) [12, 20]. In our study, the factors independently associated with 3- and 6-month mortality were in accordance with data previously published for ICU-admitted cancer patients: severity of clinical status at admission, stage of cancer, metastatic disease [12, 29, 30]. No primary site of cancer has been identified as a prognostic factor, unlike other studies that reported lung cancer as an independent predictor of hospital mortality [20]. Furthermore, the Charlson comorbidity index was not found to be associated with survival, although it is a data often found in prognostic studies in ICU [29]. Local practices may be the reason. In fact, in our study it seems that patients in most cases have few comorbidities. This probably reflects a selection at admission of patients with less comorbidity. Interestingly, we have revealed that prior chemotherapy was independently associated with a superior 3-month survival. This had not been described previously, but may be partly explained by our interest in an ICU survivor cohort. Furthermore, we have observed that the survival curves actually crossed, with a superior survival of patients pretreated with chemotherapy before 6 months and a poorer survival for those pretreated with chemotherapy thereafter.

The association between ECOG-PS of 3–4 at ICU admission and poor survival is well described. Despite a reduction in hospital mortality in the last decade, survival gain is less pronounced as the ECOG-PS worsened [19]. But very few data are available regarding the evolution of ECOG-PS after ICU stay. *Soares* et al. revealed an ECOG-PS at 6 months of 3–4 in 9.5% of hospital survivors [17]. In our study, in ICU survivors, most patients with an ECOG-PS of 0–1 at ICU admission displayed an ECOG-PS of 0–2 at 3- and 6-months. Conversely, many patients with an ECOG-PS of 3–4 at ICU admission died prior to 3 months post-discharge. No other studies have so far reported the ECOG-PS after discharge in relation with prior ECOG-PS.

Concerning anticancer treatment at 3- and 6-months after ICU discharge, we have observed that most patients with an indication for cancer treatment ICU discharge can be treated, usually with chemotherapy. Interestingly, only few patients alive at 3- and 6-months were in exclusive palliative care. However, we were unable to assess whether the implementation of palliative care was modified or delayed by ICU admission. Only very few studies have reported anticancer treatments following ICU discharge. Considering patients still alive at hospital discharge, *Soares* et al. reported that 37% of these patients benefited from anticancer treatment, such as surgical resection (7%), radiation therapy (34%), and chemotherapy (80%) [17]. In 35 (34%) patients, the initially scheduled anticancer treatment plan required either dose reduction or protocol modification. These authors also reported that poor ECOG-PS was the only factor associated with a lower probability of receiving the initially scheduled treatment plan (OR, 0.20; 95%CI 0.05–0.87; *P* = 0.032). In a smaller cancer patient cohort, 30 patients (68%) of the 44 ICU survivors with available clinical information were able to undergo a specific anticancer treatment following hospital discharge. In brief, one patient underwent surgical treatment, two received a combination of chemotherapy and radiation therapy, and 27 remaining ones were treated with chemotherapy alone [30].

One of the study strengths include the large variety of data recorded, such as data relating to ICU admission and care, cancer history before ICU to 6 months after ICU discharge with only few missing data (less than 2% for vital status at 6 months). To our knowledge, this is only the second study that specifically investigated the prognostic factors of cancer patients following ICU discharge [20], with only very few studies having reported patient characteristics following ICU discharge [21, 31, 32]. Nevertheless, there are several limitations to our study. The single-center patient recruitment limits to a certain extent the extrapolation of our results to other centers. In spite of only few missing data concerning patient characteristics at ICU admission and survival, it proved difficult to retrospectively collect the ECOG-PS at 3- and 6-months. Moreover, as the inclusion period was until 2013, we were unable to investigate patients treated with targeted therapies or immune checkpoint inhibitors.

As most of studies on critical ill cancer patients are retrospective, the impact of new anticancer treatments (targeted therapies or immune checkpoint inhibitors) or of new management of organ failure in ICU could not be evaluated. Regarding targeted therapies, main publications were case series [33, 34]. A small case-control study reported that early survival (in the first 30 days after ICU admission) was similar in patients with and without oncogenic addiction but that late survival was better in patients with mutations who were treated with targeted therapy. Regarding immune checkpoint inhibitors, an ICU admission related to immune-related adverse event was associated with better outcome. No study evaluated impact of a pre-admission treatment by immune checkpoint inhibitors on survival. Furthermore, it would be interesting to follow these patients after ICU discharge in order to report treatments received after ICU discharge.

## Conclusions

Considering the cancer patients alive at ICU discharge, 52% had an ECOG-PS of 0–2 at 3 months, while 55% benefited from an anticancer treatment. Of note, most patients with a good ECOG-PS before ICU admission displayed a good ECOG-PS following ICU discharge. These results should be taken into account when deciding upon ICU admission. At that particular time, it is paramount to have a sound concept concerning the patient’s general condition and anticancer treatment opportunities following ICU discharge. Regarding recent improvements in cancer care, it would be interesting to evaluate specifically the impact of targeted therapies and immune checkpoint inhibitors in prognosis of critically ill cancer patients.

## Supplementary Information

**Additional file 1: Supplementary Figure 1.** Estimation of survival according to previous chemotherapy (Kaplan Meier).

**Additional file 2: Supplementary Table 1.** Patient Characteristics According To Previous Chemotherapy

## Data Availability

The datasets used and/or analyzed during the current study are available from the corresponding author on reasonable request.

## Abbreviations

CI: Confidence interval
ECOG: Eastern Cooperative Oncology Group
ICU: Intensive care unit
IQR: Interquartile range
Miss.: Missing data
OR: Odds ratio
PS: Performans status
SAPS II: Simplified Acute Physiology Score
SOFA: Sequential Organ Failure Assessment
TLD: Treatment limitation decision
WHO: World health organization
