# Supplementary figures and images for: Factors associated with survival of patients with solid Cancer alive after intensive care unit discharge between 2005 and 2013
Source: BMC Cancer. 2021 Jan 5;21:9. doi: 10.1186/s12885-020-07706-3 (PMC7786972; doi:10.1186/s12885-020-07706-3)

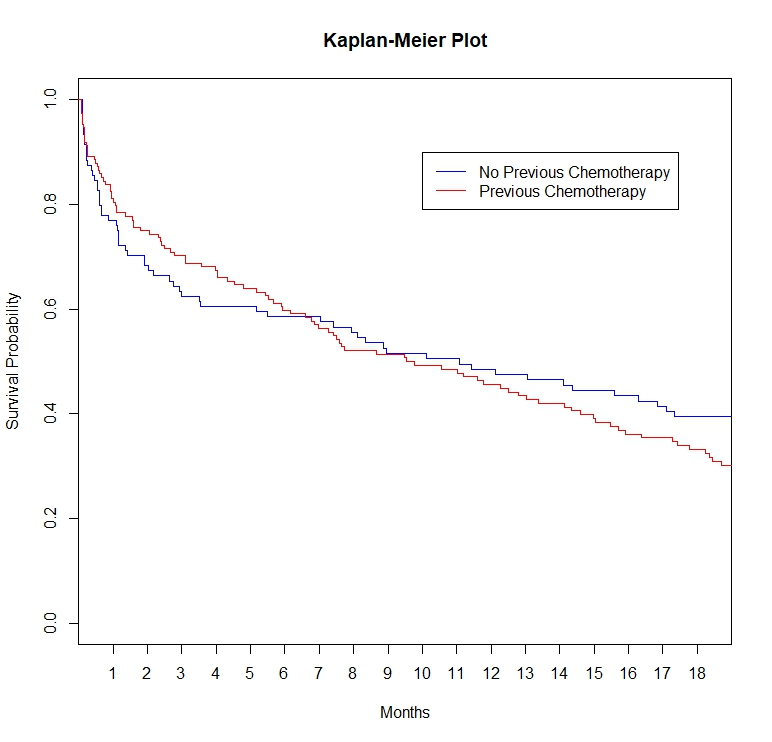

Supplement: Supplementary file 1 — Additional file 1: Supplementary Figure 1. Estimation of survival according to previous chemotherapy (Kaplan Meier). [file 12885_2020_7706_MOESM1_ESM.tif]
