# Supplementary material for: Factors associated with survival of patients with solid Cancer alive after intensive care unit discharge between 2005 and 2013
Source: BMC Cancer. 2021 Jan 5;21:9. doi: 10.1186/s12885-020-07706-3 (PMC7786972; doi:10.1186/s12885-020-07706-3)
Supplement: Supplementary file 2 — Additional file 2: Supplementary Table 1. Patient Characteristics According To Previous Chemotherapy [file 12885_2020_7706_MOESM2_ESM.docx]

**Supplementary table 1. Patient Characteristics According To Previous Chemotherapy**

| Variable | No Chemotherapy Before ICU Admission  n=104 | Chemotherapy  Before ICU Admission  n=149 | *p*-value |
| --- | --- | --- | --- |
| Survival (Kaplan Meier) | 332 (35 – 1476) | 286 (54 – 690) | 0.17 |
| Type of cancer |  |  |  |
| Digestive | 33 (32) | 46 (31) | 0.88 |
| Thoracic | 17 (16) | 48 (32) | <.01 |
| Head and Neck | 14 (13) | 18 (12) | 0.75 |
| Gynecological | 7 (7) | 22 (15) | 0.05 |
| Genito-urinary | 24 (23) | 4 (3) | <.01 |
| Metastatic disease (MD=12) | 26 (27) | 82 (56) | <.01 |
| Cancer status (MD=6) |  |  | <.01 |
| Controlled or in remission for <5 years | 39 (39) | 41 (28) |  |
| Newly diagnosed / recurrence | 48 (48) | 54 (37) |  |
| In progression | 13 (13) | 52 (35) |  |

Survivals have been expressed as median (IQR 25%-75%).
